# Brazilian adolescents’ oral health trends since 1986: an epidemiological observational study

**DOI:** 10.1186/s13104-015-1538-5

**Published:** 2015-10-12

**Authors:** Maria Vieira de Lima Saintrain, Carlos Roberto Silveira Correa, Suzanne Vieira Saintrain, Sharmênia de Araújo Soares Nuto, Anya Pimentel Gomes Fernandes Vieira-Meyer

**Affiliations:** Public Health Program, University of Fortaleza, UNIFOR, Av. Washington Soares, 1321, Fortaleza, 60811-905 CE Brazil; Public Health Program, University of Campinas-UNICAMP, Rua Tesalia Vieira de Camargo, 126 Cidade Universitária Zeferino Vaz, Campinas, 13083-970 SP Brazil; Public Health Master’s Program, University of Fortaleza, UNIFOR, Av. Washington Soares, 1321, Fortaleza, 60811-905 CE Brazil; Family Health Master Program, Oswaldo Cruz Foundation, Fiocruz, Av Santos Dumont, 5753 sala 1303 Papicu, Fortaleza, CE 60175-047 Brazil; Faculty of Dentistry, University of Fortaleza, UNIFOR, Av. Washington Soares, 1321, Fortaleza, 60811-905 CE Brazil

## Abstract

**Background:**

Oral health is part of general health, and in adolescence, it represents a good individual health indicator. Three country-based oral health epidemiological studies have been developed in Brazil (1986, 2003 and 2010). The objective of this study was to analyze oral disease trends among Brazilian adolescents and to compare these trends to the World Health Organization’s goals with a focus on public health policies implemented between 1986 and 2010.

**Methods:**

This is an epidemiological observational study performed with secondary data from Brazilian Oral Health surveys (1986, 2003 and 2010). The DMFT (number of decayed, missing and filled teeth) index was used for the 12-year-old and 15- to 19-year-old groups, and periodontal disease (CPI) and the percentage of individuals who needed and/or had prostheses were evaluated in the 15- to 19-year-old group.

**Results:**

Between 1986 and 2010, DMFT decreased from 6.65 to 2.07 (68.9 % reduction) in the 12-year-old group and from 12.68 to 4.25 (66.5 % reduction) in the 15- to 19-year-old group. In all groups, the missing component had the strongest decrease. Adolescents had a reduction of 20.3 % in access to dental care. In 2003, in the 15- to 19-year-old group, 89.5 % of teenagers had at least one decayed tooth, while in 2010, the value was 76.1 %. In 2010, the percentage of adolescents without gingival problems varied among different regions of Brazil, with 30.8 % in the North and 56.8 % in the Southeast. Regarding DMFT, the difference between the North and Southeast Regions was 84 %.

**Conclusions:**

Improvement trends regarding adolescent oral health were observed, which seem to be supported by health education and promotion activities along with the reorganization of the Brazilian health system.

## Background

Oral health is part of general health, and in adolescence, it represents a good individual health indicator [1]. Studies regarding dental decay showed a decrease in poor oral health worldwide in this population. In 12-year-old children, the DMFT (number of decayed, missing and filled teeth) score declined from 4.32 to 3.20 between 1995 and 2003 in Poland; in China, it decreased from 1.03 to 0.5 between 1995 and 2005 [2, 3]. The same trend was found in Japan, where the incidence of dental caries in young adults decreased between 1957 and 2005 [4].

In Brazil, a countrywide oral health epidemiological survey performed in 1986 aimed to determine the prevalence of major dental problems in the urban populations of 16 Brazilian cities and to provide subsidies for the implementation of an oral health program [5]. This study reported the poor oral health conditions of adolescents, with a DMFT value of 6.65 at age 12 and 12.68 in the group between 15 and 19 years of age.

The Federation Dentaire Internationale (FDI, World Dental Federation) and the World Health Organization (WHO) [6] considered a DMFT of up to three per person at age 12 as “acceptable” in 2000. According to the same institutions, 85 % of the population should retain all their teeth at the age of 18 years in 2000. For the year 2010, it was proposed that the DMFT value should be equal to one at age 12 with no tooth loss at age 18. Regarding gingival bleeding and dental tartar, the recommendation was no more than a sextant affected in children at age 15 [7].

Between 2002 and 2003, the project “SB Brazil—Oral Health Conditions of the Brazilian Population-2003” was developed to generate information on the oral health of the Brazilian population and to subsidize the planning and evaluation of actions in this area in different levels of management of the Brazilian National Health System [8]. This survey had a population larger than the first one; however, both had comparable sampling and clinical evaluation methodologies.

In 2010, another dental survey [9] was carried out, with the results were published in 2011. This study was developed in 177 cities, covering all states/regions of Brazil, and used the same methodology as the previous studies. The three country-based epidemiological studies (1986, 2003, and 2010) represent a historical process that enables, for the first time, data regarding oral health to be compared and dental decay trends to be analyzed. The hypothesis is that the health policies implemented during this period improved adolescents’ oral health. An understanding of oral health behavior in adolescents is relevant for oral health teams because it reveals which health promotion activities are needed at different levels in this age group.

This study aimed to analyze the oral disease (dental caries and periodontal problems) trends among Brazilian adolescents during the period of the epidemiological surveys, from 1986 to 2010. Trends analyses, such as those considering the total population of a country, are important because they help health planning and decision-making [10].

## Methods

This study analyzed the oral disease behavior in Brazilian adolescents, which is classified as the 10- to 19-year old age group according to the World Health Organization [11]. In this study, we compared Brazilian populations at different times.

To compare the epidemiological situations, secondary data, through oral health indicators, were obtained in the 1986, 2003 and 2010 studies: the Brazil urban areas in 1986, the SBBrazil-2003 Project and the SBBrazil-2010 Project [5, 8, 9]. It is important to note that despite using the same index to measure the presence of caries (DMFT) for the 1986 epidemiological survey, the methodological rigor was higher due to the use of a dental probe to detect caries in proximal surfaces, sulci and fissures [5]. This rigor was lacking in the last two surveys because of the remineralization process of tooth enamel and, in particular, to avoid potential iatrogenic dental caries caused by the use of the explorer probe.

For the 12-year-old group, the DMFT index for permanent dentition was considered. For the 15- to 19-year-old group, the DMFT index, periodontal disease (CPI), the percentage of adolescents with all healthy sextants, gingival bleeding, dental calculus, periodontal pockets, and the percentage of individuals who needed and/or had prostheses were considered [5, 8, 9].

The indexes of caries experience in permanent teeth (DMFT) and periodontal disease (CPI) were evaluated according to the World Health Organization [12] recommendation. In all reference studies, the examiners were trained and calibrated [5, 8, 9].

To analyze the trends in dental caries (DMFT, decayed, missing and filled teeth) in the 12-year-old and 15- to 19-year-old groups, the statistical package SPSS 20.0 (Statistical Package for Social Science Inc., Chicago, IL, USA) was utilized.

There was no need for an evaluation by the Research Ethics Committee because this study used data in the public domain and did not have a conflict of interest. Nevertheless, it is important to mention that the original epidemiological studies followed all ethical norms at the time of the study, including obtaining signatures for informed consent from those participating in the survey.

## Results

For both age groups studied in this manuscript, DMFT was evaluated in a total of 6590 individuals in the 1986 epidemiological study, 51,833 in the 2003 study, and 12,614 in the 2010 study.

In 1986, 32 % of 18-year-old participants had all their teeth; in 2003, this percentage was 55.1 %, and in 2010, the percentage was 86.3 % [5, 8, 9]. Increases in dental calculus and periodontal pockets were observed in the 15- to 19-year-old group; in 2010, only 50.9 % of the observed adolescents presented with all healthy sextants.

A trend of DMFT improvement was observed for the research period (1986–2010); the DMFT decreased by 68.9 % at age 12 and by 66.5 % for the 15- to 19-year-old group (Table 1). The data from 2003 showed that in the age group between 15 and 19 years, 89.5 % of teenagers had at least one decayed tooth, whereas in 2010, the value was 76.1 %.

**Table 1:** Dental care trend considering the components of DMFT Index in adolescents

Component DMFT Index													
Variable		Year 1986		Year 2003		Year 2010		(2003–1986)^a^	2003–1986^b^	2010–2003^a^	2010–2003^b^	(2010–1986)^a^	2010–1986^b^
		Value	%^c^	Value (SD; CI 95 %)	%^c^	Value (CI 95 %)	%^c^						
12 years													
Decayed		3.65	54.9	1.69	60.8	1.21	58.5	−1.96	−53.7	−0.48	−28.4	−2.44	−66.8
Missing		0.88	13.2	0.18	6.5	0.12	5.8	0.70	−79.5	0.06	−33.3	0.76	−86.4
Filled		2.12	31.9	0.91	33.7	0.73	35.3	1.21	57.1	0.18	19.8	−1.39	−65.6
Total	Brazil	6.65	100.0	2.78	100.0	2.07	100.0	−3.87	−58.2	−0.71	−25.5	−4.58	−68.9
	North	7.50		3.13 (3.13; 3.05–3.21)		3.16 (2.55–3.76)		−4.37	−58.27	0.03	0.96	−4.34	−57.87
	Northeast	6.90		3.19 (3.57; 3.11–3.27)		2.63 (2.02–3.24)		−3.71	−53.77	−0.56	−17.55	−4.27	−61.88
	Central-west	8.52		3.16 (3.30; 3.08–3.24)		2.63 (2.14–3.13)		−5.36	−62.91	0.53	−16.77	−5.89	−69.13
	Southeast	5.95		2.3 (2.72; 2.24–2.36)		1.72 (1.36–2.08)		−3.65	−61.34	−0.58	−25.22	−4.23	−71.09
	South	6.31		2.31 (2.71; 2.25–2.37)		2.06 (1.66–2.45)		−4	−63.9	−0.25	−10.82	−4.25	−67.35
15–19 years													
Decayed		4.26	233.6	2.79	45.2	1.70	82.1	−1.47	−34.5	−1.09	−39.1	−2.56	−60.1
Missing		2.52	19.9	0.89	14.4	0.38	18.4	−1.63	−64.7	−0.51	−57.3	−2.14	−84.9
Filled		5.88	46.4	2.49	40.4	2.16	104.3	−3.39	−57.7	−0.33	−13.3	−3.72	−63.3
Total	Brazil	12.68	100.0	6.17	100.0	4.25	205.3	−6.51	−51.3	−1.92	−31.1	−8.43	−66.5
	North	8.93		6.14 (4.79; 5.99−6.29)		5.64 (5.06–6.23)		−2.79	−31.24	−0.5	−8.14	−3.29	36.84
	Northeast	12.99		6.34 (4.91; 6.19–6.49)		4.53 (4.04–5.01)		−6.65	−51.19	−1.81	−28.55	−8.46	−65.13
	Central-west	10.43		6.97 (5.12; 6.75–7.19)		5.94 (5.20–6.69)		−3.46	−33.17	−1.03	−14.78	−4.49	−43.05
	Southeast	18.64		5.94 (4.66; 5.77−6.11)		3.83 (3.23–4.43)		−12.7	−68.13	−2.11	−35.52	−14.81	−79.45
	South	9.84		5.77 (4.62; 5.62–5.92)		4.01 (3.35–4.67)		−4.07	−41.36	−1.76	−30.50	−5.83	−59.25

Brazil, 1986/2003/2010

A total of 1792 12 years old were evaluated in 1986, while in 2003 there were 34,550 and in 2010, 7247. Regarding the 15–19 years old, in 1986, 4798 were evaluated, in 2003 16,833, and in 2010 5367

Source: Brazil. MS, 1986; 2004, 2011

^a^Represents the difference of the mean value obtained for each year

^b^Represents the percentage of the difference (positive or negative) of the mean value

^c^Represents the percentage of each DMFT component (decayed, missing and filled) on the total value of the DMFT Index

Tables 1, 2, 3 and 4 present the specific trends among the three epidemiological studies for a series of findings related to caries experience, periodontal status, usage or need of complete prosthesis, and access to dental care. For DMFT for the 12-year-old and 15- to 19-year-old groups (Table 1), the data are described by the mean. When the results are shown by region, the 1986 data are presented as the mean, and the 2003 data are present as the mean, standard deviation and 95 % confidential interval. The mean and 95 % confidential interval are presented for the 2010 data. The differences in the data formats are related to the format in which the information was originally available. In Table 2, the periodontal condition is described as the mean for 1986 and 2003, but for 2010, the data are described as the mean and 95 % confidential interval. In Tables 3 and 4, the values are presented as the mean per year.

**Table 2:** Percentage of individuals and trend according to the periodontal condition−CPI Index—1986/2003/2010

Periodontal condition—CPI index									
Variable	Year 1986 n = 4.798	Year 2003 n = 7.772	(2003–1986)	Trend (%) 2003–1986	Year 2010 n = 5.445	(2010–2003)	Trend (%) 2010–2003	(2010–1986)	Trend (%) 2010–1986
15–19 years					Value (CI 95 %)				
Healthy	51.68	46.18	−5.5	−10.7	50.9 (45.4−56.4)	4.73	10.2	−0.78	−1.5
Bleeding	19.89	18.77	−1.1	−5.6	9.7 (7.5–12.3)	−9.07	−48.3	−10.19	−51.2
Calculus	23.10	33.40	10.3	44.6	28.4 (24.8–32.4)	−5	−15.0	5.30	22.9
Pocket 4–5 mm	2.14	1.19	−1.0	44.4	8.8 (6.9–11.3)	7.61	639.5	6.66	311.2
Pocket 6 mm and +	0.19	0.15	−0.04	−21.1	0.7 (0.3–1.2)	0.55	366.7	0.51	268.4
Excluded sextant	2.92	0.31	−2.6	−89.4	1.5 (0.9–2.4)	1.19	383.9	−1.42	−48.6

Source: Brazil. MS, 1986; MS, 2004, 2011

**Table 3:** Percentage of individuals who need and/or have total dental prostheses

Age	Year 1986 %	Year 2003 %	(2003–1986)	Trend (%) 2003–1986	Year 2010	(2010–2003)	Trend (%) 2010–2003	(2010–1986)	Trend (%) 2010–1986
15–19 years	1.73	0.04	−1.69	−97.7	0.00	−0.04	−100.0	−1.73	−100.0

Source: Brazil. MS, 1986; MS, 2004, 2011

**Table 4:** Percentage of adolescents who had access to dental care in the last year

Dental care in the last year									
Age	Year 1986 %	Year 2003 %	(2003–1986)	Trend (%) 2003–1986	Year 2010	(2010–2003)	Trend (%) 2010–2003	(2010–1986)	Trend (%) 2010–1986
15–19 years	67.59	86.57	19.0	28.1	53.9	−32.67	−37.7	−13.69	−20.3

Source: Brazil. MS, 1986; MS, 2004, 2011

It is important to note that in the 2003 epidemiological study, a difference of DMFT in areas with fluoridated versus non-fluoridated water was observed. DMFT in the 12-year-old group was 2.27 in fluoridated areas and 3.38 in non-fluoridated areas; for the 15- to 19-year old group, the DMFT was 5.69 in fluoridated areas and 6.56 in non-fluoridated areas [8].

In 2010, the SBBrasil-2010 Project [9] observed that the percentage of adolescents without gingival problems varied among different regions of Brazil, from 30.8 % in the North Region to 56.8 % in the Southeast Region. Regarding the DMFT, the difference in the value between the North and Southeast Regions was 84 %. In addition, the proportion of filled teeth, which is related to the overall DMFT value, was smaller in the Northeast Region than in the Southeast Region.

Utilizing linear regression, a significant exponential decrease in DMFT for the 12-year-old group was found using the equation DMFT = 1.55E+43e^year (r = 0.998; r^2^ = 0.996; p = 0.027). This may be explained by the decayed (D) component, which also had an exponential decrease (D = 1.34E+41^year; r = 0.999, p = 0.008). Regarding the 15- to 19-year-old group, there was a linear decrease in the DMFT prevalence during the study period (DMFT = −0.357 year+721.87; r^2^ = 0.997; p = 0.044) as well as a decrease in the missing (M) component (M = −0.09 year + 182.06). Utilizing these equations, one can verify that the DMFT for the 12-year-old group would be 1 (as proposed by the WHO for 2010) in 2029.

## Discussion

This is the first time that a trend study was performed evaluating the 1986, 2003 and 2010 epidemiological data (24-year period) in the five geographical regions of Brazil. Epidemiological studies contribute to the identifications of the magnitude of a problem and can be used to plan and evaluate public policies, whether with an educational, preventive or rehabilitative nature.

A decrease in the prevalence of dental caries in both age groups (12 and 15–19 years) was observed. Regarding the DMFT components, the proportion of decrease in both age groups was similar; however, the reduction was more evident in the missing component of the index. When comparing this reduction to the values recommended by the WHO/FDI for 2000 [7], only the 12-year-old group achieved these values by 2003 (DMFT) [8]. Regarding the WHO/FDI “accepted” values for 2010, according to the equation created in this study, the DMFT goal for the 12-year-old group [1] would not be achieved until 2029.

Regarding the “accepted” values for 2010, the goal of no tooth loss for 18-year-old adolescents was not achieved. Although a decrease in the percentage of adolescents who needed dental prosthesis was observed between 1986 and 2010, partial edentulism was still present in this age group, and this issue may adversely affect health by causing a nutritional deficiency due to the decrease in mastication capacity [13], among other social problems. Nevertheless, improvements can be observed in this field, especially considering that in 1986, only 32 % of 18-years-olds had all their teeth and that in 2010, this value had increased to 86.3 % [5, 9]. The most plausible explanatory hypotheses for the decline in DMFT among Brazilian adolescents are the increase in access to fluoridated water and toothpaste in the studied population and changes in public oral health programs [10].

According to Natarajan [14], the global DMFT average is 1.67 for 12-year-olds, which indicates that the values found in Brazil (DMFT of 2.07) are above the world average. Considering that Brazil is a country with strong developmental rates in most socio-economic areas, it is clear that public policies have not achieved their goals regarding the population’s oral health, and much work must be performed to improve in this area. Nevertheless, the decrease in the caries index was of a good magnitude, which prompts an interesting discussion of how oral health public policies unfolded in the country and how they contributed to the DMFT values observed in this study. The analysis of oral disease trends among Brazilian adolescents may contribute to an assessment of the Brazilian health system. According to Araujo [15], this evaluation enables the verification of past decisions (accuracies and errors) and a discussion of the future.

Four public oral health actions that affected the general population can be highlighted in modern Brazilian history, and these may have influenced the reductions observed in the studied groups. The first action was the fluoridation of the water supply, the second was the insertion of fluoride in toothpaste, the third was the structuration of the oral health primary care system, with the creation of an oral health team as part of the family health team (a nationwide policy for primary health care), and the fourth was the creation of a Nationwide Oral Health Policy entitled ‘Brasil Sorridente’ (Smiling Brazil) [16].

In 1974, federal law 6050 decreed that every new water treatment plant in the country had to install a fluoridation unit and that all renovations in existing water treatment plants had to include a fluoridation unit [17]. With this regulation, part of the water supply of the country became gradually, albeit slowly, fluoridated over the years. To understand the magnitude of this policy, one can examine the 2003 epidemiological survey data (29 years after federal law 6050), which noted that only 46 % (n = 115) of the 250 cities evaluated had a fluoridated water supply. Moreover, there was an uneven distribution of the fluoridated cities; such cities were concentrated in the municipalities of the South and Southwest Regions as well as in the more populated cities. Only 6 % (n = 3) of the cities in the North Region and 16 % (n = 8) of those in the Northeast Region had a fluoridated water supply, whereas in the South, Southeast and Central-west Regions, the values were 66 % (n = 33), 88 % (n = 44) and 54 % [18], respectively [8]. According to Frias et al. [19], the prevalence of dental caries reflected determinant factors of a biological, dietary, behavioral and socio-economic nature as well as factors of access to goods and healthcare services. Knowing that those areas have a higher socio-economic status compared to the North and Northeast parts of the country and therefore a lower caries risk, one must relativize the difference in DMFT among fluoridated and non-fluoridated areas. It is quite obvious that this difference is mostly explained by the fluoridation of the water supply; however, other factors, such as socio-economic status, could have, and probably did, influence the DMFT in these distinct areas.

In December of 1989, through ordinance 22 of the Health National Office, toothpaste fluoridation was regulated in Brazil. The levels were established for adult toothpaste (between 1000 and 1500 ppm) as well as for pediatric toothpaste (between 500 and 750 ppm) [20]. Data regarding toothpaste usage in Brazil are limited, and this usage depends on a series of factors, such as culture and access to toothpaste, which may vary between regions. A study in Guaiúba-CE (Northeast Region of Brazil) observed that 93.4 % of adolescents use toothbrushes and 89.9 % use toothpaste [21]. Considering that Guaiúba is a municipality without water fluoridation, one can perceive the importance of this public action as well as the dissemination of topical fluoride usage through fluoridated toothpaste.

The third oral health publication happened as a consequence of the restructuration of the public health system in Brazil—the Unified Health System (UHS). This system was created in 1988 with the promulgation of the new constitution of the country; however, in 1990, it was regulated by federal laws 8080 and 8142. A long process based on the principles of universality, equity and integration has since taken place. For primary care, a Family Health Program (created in 1994) was organized, in which a doctor, nurse, nurse assistant and health agents form the family health team responsible for taking care of a delimitated number of persons (approximately 1000 families) in a specific area. In 2000, the Federal Government (Ministry of Health—ordinance 1.444) included an oral health team, including a dentist and an assistant, in the family health team [22]. With this action, access to oral health care increased. In 2002, there were 4261 oral health teams, and by 2010, the number of teams increased to 20,300 [23]. The oral health team redirected the work process emphasizing water fluoridation, reorganizing primary care, especially the Family Health Program, and reorganizing the specialized oral health service [16, 24].

As a result of this program, a series of improvements were observed in the oral health care system between 2002 and 2010: the 2008 publication of guidelines for oral health attention in primary care [25]; the installation of 600 new fluoridation units in 434 cities distributed in 11 states, which benefitted 7 million individuals; an increase in the number of oral health teams in the country (from 4261 to 20,300); the distribution of 72 million oral health kits (toothbrush and toothpaste); the installation of prosthetic laboratories in 664 municipalities; and the creation of Specialized Oral Health Centers—SOHC (a total of 853) [23]. The SOHC are responsible for treatments covering endodontics, special need patients’ care, dental surgeries, periodontal treatment, prosthetic services, etc. Because endodontic treatment was not a reality in public service before Smiling Brazil, one can argue that, together with health promotion actions and early dental caries treatment, it helped decrease the number of extractions (missing teeth component) in the DMFT index observed over the last few years.

It is important to note that due to the Smiling Brazil actions, the access to dental care increased three-fold between 2003 and 2008 [23] for the general population; however, as can be observed in Table 4, the inverse trend occurred among teenagers in this study, with a decrease of 37.7 % in dental consultations between 2003 and 2010. The reason for this is not clear, and further investigation on this subject is needed. One possibility is that before the unified health system was started, the government actions were focused on specific groups, such as children and adolescents [26]. With the universalization of the service, access to the general population increased; however, due to the repressed demand of the other groups, individuals who were initially prioritized could have experienced a decrease in access due to care provided to previously non-prioritized groups.

Researchers [27] in New Zealand reported that patients with frequent dental care visits present with better self-rated oral health conditions, less dental decay and less tooth loss than those without frequent dental visits. This study supports the belief that routine dental care is associated with good oral health, and therefore, this practice should be incentivized [18, 28]. The present study showed that actions/policies regarding Health Promotion, Health Education and Health Care that are based on intersectorial collaboration and interdisciplinarity can positively influence the oral health status of a nation. Nevertheless, it is important to note that further developments in this area are needed to guarantee good levels of oral health for Brazilian adolescents, as this is still a public health problem in Brazil.

Nevertheless, it is important to note that this study was based on a secondary data analysis, which limits the statistical analysis of the results. It is also necessary to mention that there are differences when comparing the three epidemiological studies, including the design and implementation of the surveys.

Lastly, it is important to note that studies such as the present one are important for providing information for and analyses of the organization of future oral health goals. According to the authors of “Global goals for oral health 2020” [29], a publication based on the efforts of a working group with representatives from the FDI, WHO and IADR, there is a range of possible areas that need to be considered when plans are being developed. According to these authors, the goals should be established based on local circumstances (epidemiological, political, socio-economic, cultural and legislative context), which is what we tried to analyze in the present work.

Overall, the present study observed improvements in adolescents’ oral health, which might have been supported by health education and health promotion actions along with the reorganization of the Brazilian health system. However, this issue remains a public health problem in Brazil and deserves further studies and public policies regarding its improvement.
